# Measuring Exposure in Hurricane Katrina: A Meta-Analysis and an Integrative Data Analysis

**DOI:** 10.1371/journal.pone.0092899

**Published:** 2014-04-08

**Authors:** Christian S. Chan, Jean E. Rhodes

**Affiliations:** 1 Department of Psychology, The University of Hong Kong, Hong Kong SAR, China; 2 Department of Psychology, University of Massachusetts Boston, Boston, Massachusetts, United States of America; University of California, San Francisco, United States of America

## Abstract

To date there is no consensus on the operationalization of exposure severity in the study of the impact of natural disasters. This is problematic because incomplete and inconsistent measurement of exposure limits the internal and external validity of disaster studies. The current paper examined the predictive validity of severity measures in two interrelated studies of Hurricane Katrina survivors. First, in a meta-analysis of eight studies that measured both exposure severity and posttraumatic stress, the effect size was estimated to be *r* = .266. The moderating effects of sample and study characteristics were examined and we found that minority status and number of stressors assessed were significant moderators. Second, in an integrative data analysis of five independent samples of Hurricane Katrina survivors, the impact of specific disaster-related stressors on mental health was compared. Threat to physical integrity of self and others were found to have the strongest association with posttraumatic stress (PTS) and general psychological distress (GPD). The lack of basic necessities, such as food, water, and medical care, and loss of pet were also found to be strongly associated with both PTS and GPD. The results from the two studies are integrated and their implication for disaster research and relief are discussed.

## Introduction

Natural disasters can be profoundly and pervasively disruptive [1]–[3]. Reviewing the literature, Norris and colleagues [2] estimated the overall prevalence rate of severe and very severe psychological impact after a natural disaster at around 34%. In another review [4], found that the prevalence rate of PTSD ranged from 5% to 60%.

Exposure is considered one of the key predictors of psychological outcomes in disasters in both adults [2], [4]–[6] and children [7]. Nonetheless, variations in disasters as well as the measurement of disaster-related stressors have complicated our understanding of the association between disaster exposure and outcome. First, there is considerable heterogeneity in survivors' disaster response, both within and between disasters. Past meta-analyses have found a large degree of heterogeneity in effect sizes of risk factors across studies [8], [9]. Such heterogeneity is a combination of systematic variability in survivors' experiences and error. Even in the context of the same disaster type, survivors can experience very different levels of exposure [2], [4]. Moreover, the cultural and community contexts in which disasters occur further complicate the comparability and generalizability between studies.

Second, there is a lack of consensus on the measurement of the construct. Disaster-related stressors (DRS) have been assessed in a variety of ways, including inquiring about the loss of life and bereavement, threat to life, injury, fear, witnessing injury and death, property damage and financial loss, loss of social and personal resources, as well as stressors related to relocation and chronic stressors after the disaster [10]. Many studies use a combination of these DRS in their operationalization of exposure, often including both “objective events,” such as injury, death, or property loss, and “subjective experience,” such as life-threat [11], [12]. These DRS are often aggregated into a checklist to create a composite severity scores. Unfortunately, there has been no consensus regarding the number items to be included on such check-lists. Responding to the lack of consistency in the measurement of exposure severity, a few assessment tools have been developed, such as the Traumatic Exposure Severity Scale (TESS) [13] and the Hurricane Related Traumatic Experiences (HURTE) [7]. Both scales and their variants have been found to be associated with mental health outcomes and both have been used in a number of studies across different disaster events but have not been adopted as standards. The lack of consensus on the measurement of the construct makes it difficult to assess the impact of DRS or to compare their impact across samples or study characteristics. With these challenges in mind, the present study quantitatively synthesized primary disaster studies in order to estimate the impact of severity of exposure on symptoms.

Two interrelated studies of Hurricane Katrina survivors were conducted. First, a meta-analysis was conducted to summarize the effect size of the association between exposure severity and posttraumatic stress (PTS). Second, in an integrative data analysis—a method of combining raw data from multiple samples—of Hurricane Katrina survivors, the impact of specific disaster-related stressors on mental health was compared.

## Study 1: Meta-Analysis

### Introduction

Although it is difficult to disentangle the effects of different sources of variation on individual survivors' symptoms, meta-analysis offers a way to account for between-study variability, especially differences in study design and sample characteristics. By focusing on one major disaster—Hurricane Katrina—we limit the systematic variability between studies to the differences in sample and study characteristics that might moderate the relationship between exposure severity and PTS. We also considered the moderating effect of both sample composition (i.e., gender, age, race, neighborhood) and study characteristics (e.g., timing of assessment, number of items) on the association between measures of exposure and PTS. Eliminating between-disaster variability allows us to focus on the influence of operationalization of “exposure” on study outcomes, which is our prime research question.

#### Moderators: Sample Characteristics. Gender

Women are at significantly greater risk than men for post-disaster psychopathology, including posttraumatic stress, anxiety and depression [8], [14], [15]. Women are also relied upon more than men in the aftermath of natural disasters [16], [17], which may leave them less able to attend to their own psychological needs, putting them at greater risk of post-disaster psychological dysfunction. Indeed, compared to their male counterparts, female survivors of Hurricane Katrina have reported more PTSD and mental health symptoms [18]. Hence, we hypothesized that gender ratio within samples would moderate the relationship between exposure severity and PTSD symptoms, with stronger associations seen in samples in samples with a higher percentage of women.

#### Race and Ethnicity

Members of minority communities are at particularly high risk of poor physical and mental health in general [19], [20]. Census data indicated that 67% of the residents of the city of New Orleans before Katrina were Black, about a third of whom lived below the poverty line [21]. Consistent with media reports during the aftermath of Katrina, researchers have documented that the hurricane had a greater impact on Black communities than on White communities, particularly in the city of New Orleans [22]. Blacks were less likely to have an evacuation plan in place prior to the storm [23], and were less likely to evacuate prior to the hurricane [24], increasing their risk of exposure to the storm. Racial disparities in economic outcomes of Katrina survivors are also evident in unemployment rates [24], as well as in reports of difficulties accessing healthcare and of general life disruption. Blacks reported greater levels of stress than Whites in the aftermath of Katrina [24], and greater levels of anger and depression [25]. Similarly, there is evidence to suggest that immigrants and minority groups are often worse off in the aftermath of natural disasters, compared to their counterparts in majority groups [26]. We hypothesized that the minority ratio within samples would moderate the relationship between exposure severity and PTSD symptoms, with stronger associations seen in samples with a higher percentage of minority participants.

#### Age

Both younger and older ages have been found as a risk factor for PTSD in some studies [27] and not others [28]. In this meta-analysis, we included only primary studies that used an adult sample (i.e., age 18 and above). Given the inconsistency in the literature, we did not have a specific *a priori* hypothesis about age as a moderator.

#### Moderators: Study Characteristics

In addition to sample composition, study characteristics can also moderate the relationship between exposure severity and PTS. For example, Ozer et al. [9] highlighted the importance of methodological differences between primary studies, especially the timing of measurement, when accounting for discrepancies between their own meta-analysis and that of Brewin et al. [8].

#### Timing of measurement

Since the impact of a single traumatic event typically dissipates over time [2], we hypothesized that studies that were conducted soon after the Hurricane would show stronger associations between exposure severity and PTS.

#### Number of exposure items

The event checklist approach to assess exposure severity is limited by the type and number of DRS included on the list. As Netland [29], [30] has argued, such checklists ought to be as comprehensive as possible to ensure the each relevant stressor is captured and accounted. Since there is no consensus on the number of DRS to assess, it would be of interest to examine whether the number of items moderates the strength of association between exposure severity and PTS. Although no studies to date have isolated the effects of item number, it would seem logical that the larger the number of DRS, the more variability there will be in the composite score, which in turn might help explain more variance of the outcome variable.

#### Study location

According to the 2010 census data, the city of New Orleans was 29 percent less populated than it was in 2000 [31]. Although if the exact percentage of residents who have returned to the storm-affected region remains unclear, it can be inferred that a considerable portion of former residents are still displaced. A number of studies on Hurricane Katrina survivors were conducted outside of the Gulf Coast region, with those who relocated to a different city or state. Although there were no a prior studies to guide predictions, it was of interest to examine whether the relocation status moderates the strength of the relationship between exposure severity and PTS.

### Methods

#### Literature Search

Relevant studies were identified via PsycINFO and PubMed searches for materials published from 2005 (the year Hurricane Katrina occurred) to December 2011. The following keywords were entered in various combinations: *Hurricane Katrina*, *stress*, *distress*, PTS*, *PTSD*. Searches were limited to studies that were peer reviewed, written in English, and sampled from adult populations (age 18 years and older).

All manuscripts obtained with the searches were read to determine whether both exposure and symptoms of PTSD had been assessed. Studies on responders, rescue workers, and volunteers were excluded, as were treatment studies. Because we were interested in the relationship between the severity of exposure and PTS, the selection of studies was limited to those that quantitatively measured both variables, and, in addition, reported their bivariate relationship. In cases where multiple studies were published from the same data, one study that provided the relevant statistics for effect size calculation was chosen. When more than one study met all the requirements, the one with the largest sample size was used. These procedures yielded eight independent, empirical studies, which were included in the current meta-analysis ([32]–[39]).

#### Procedure. Calculation of effect sizes

One effect size (ES) was extracted from each study based on the correlation between severity of exposure and PTS. Following the procedures described by Rosenthal [40], the correlation coefficients were then converted into Fisher's *zr*, which were then used for all analyses and were weighted by their degrees of freedom (*n* - 3) in order to take into account the differential precision of estimate associated with different sample sizes. Finally, the Fisher's *zr* was converted back to *r* to yield a weighted average ES. Higher values of *r* indicate a stronger positive association between exposure severity and PTS. We used Cohen's [41] guidelines for interpreting the size of sample-weighted average correlations: .10, .30, and .50 correspond to small, medium, and large ES, respectively.

#### Heterogeneity

To test for heterogeneity of the effects of exposure severity across the studies, and the extent of it, *Q* and *I* ^2^ statistics were used. A significant *Q* value for homogeneity indicates a heterogeneous set of studies [42]. That is, variation in the true effect sizes exists. On the other hand, *I* ^2^, which ranges from zero to 100%, is the proportion of the observed variance that reflects actual differences in ES across studies [43].

#### Fixed-effect vs. random-effect models

Fixed-effect and random-effect models are two of the most common statistical models in meta-analysis, each with its own sets of assumptions and varying degree of generalizability. Because we were interested in drawing inferences that can be generalized to a larger population of survivors of Hurricane Katrina, the random-effects models was chosen to calculate the mean of *zr* and 95% confidence limits [40].

#### Publication bias

Because studies with higher effect sizes are more likely to be published than their counterparts with smaller effect sizes, a synthesis of published studies might lead to biased results. Three methods were used to address this potential problem: visual examination of a funnel plot [44], Egger's regression test [45] and Duval and Tweedie's trim-and-fill procedure [46]. First, a funnel plot of the effect sizes plotted by the standard error was created. Asymmetry in the funnel plot suggest that the existence of publication bias. Egger's regression test was performed to assess whether the funnel plot's asymmetry was statistically significant. Next, the trim-and-fill procedure was performed to provide an estimation of the number of missing studies to be added to create a more symmetric funnel plot and to estimate the impact on the ES of including the imputed studies in the synthesis.

#### Moderator Analysis

In addition to ES estimation, the study also examined whether, and to what extent, sample and study characteristics moderated the ES. The six characteristics that were considered included average age of participants, percentage of female participants, percentage of minority in terms of race and ethnicity, timing of assessment, number of items included in the exposure measure, and study location. Minority status was based on the percentage of non-white and Hispanic participants. Timing of assessment was coded in terms of the number of months since the onset of Hurricane Katrina when the study was conducted. When a range of months was given, the middle of the range was taken. Study location was dummy-coded for whether or not it was conducted in an affected region. The moderating effects of each of these characteristics were examined independently using random-effects meta-regression analysis, estimated with maximum likelihood. All analyses were conducted using *Comprehensive Meta-Analysis* [47].

### Results

#### Study selection

**Figure 1:**
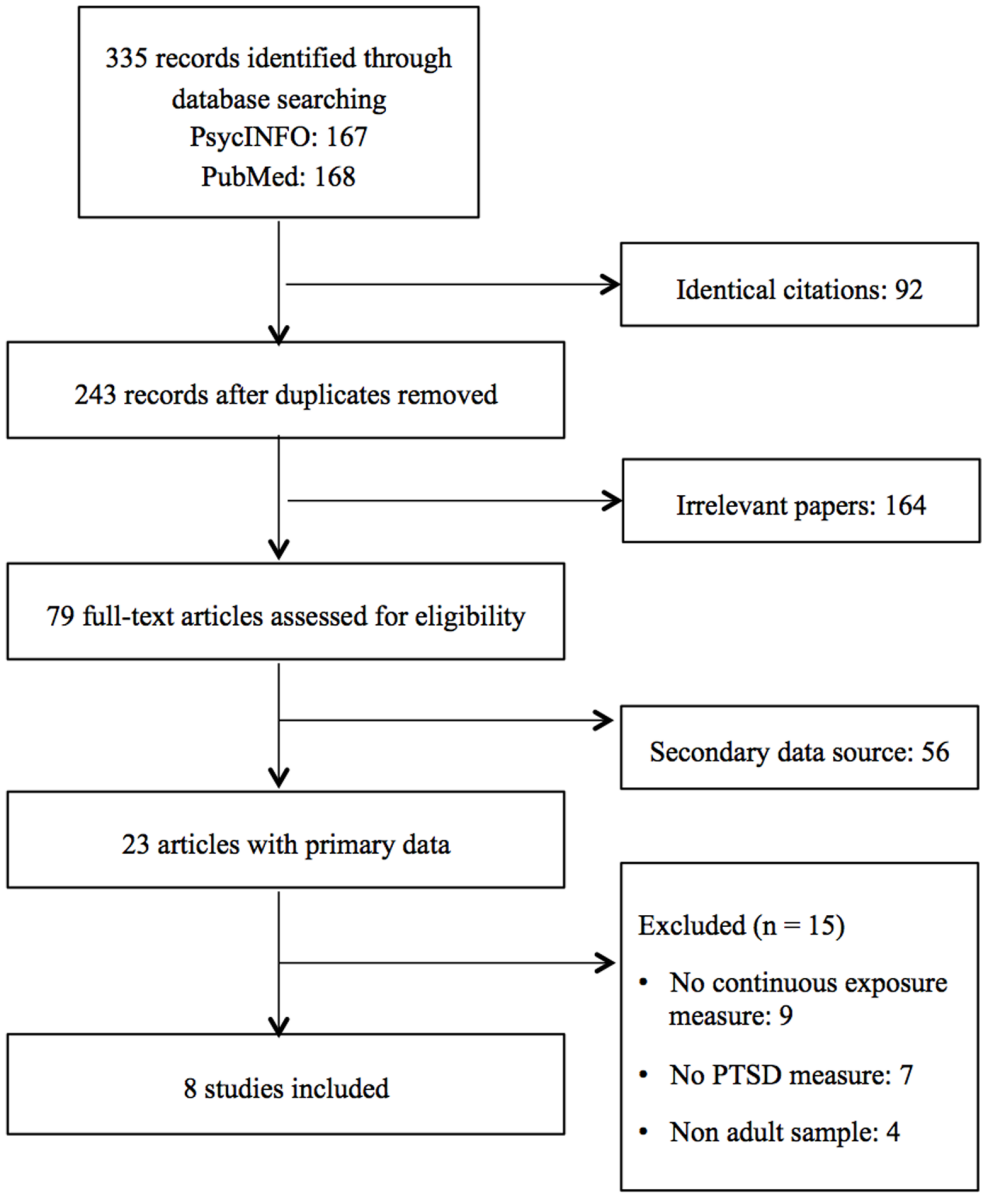
Flowchart of study identification.

The search yielded 167 studies, of which 93 were irrelevant to this review. Seventy-four articles were retrieved in full-text. Sixty-eight articles were excluded for the following reasons: studies that used secondary data, had no continuous measure of exposure, had no PTSD measure, or were conducted with non-adult samples (Figure 1). The remaining eight studies met the inclusion criteria.

#### Characteristics of the Articles

Sample and study characteristics are described in Table 1. Across the eight studies, sample size varied from 90 to 968, with a mean of 366.75 (*SD* = 303.29) and median of 343.50. The aggregated sample size was 2,934. All eight studies included a mixed gender sample, with women slightly more represented, mean = 53.5% (median = 59.2%, range = 33.3–72.74%). Seven studies provided some information on participants' race and ethnicity. One study consisted of only African American participants, and the remaining six studies had different proportions of minority (nonwhite, including Hispanic) participants, ranging from 7 to 98.6%. The mean ages of the participants of the eight studies ranged from 20.7 to 54.0 years old, with an overall mean of 39.6 and median of 41.8. The studies indicated specific populations: Cieslak et al. [34], included participants who were HIV positive prior to Katrina, Cepeda et al. [33] recruited participants who used illicit substances after the storm, and the sample in Reuther et al. [36], were college students.

**Table 1:** Study characteristics, sample characteristics and weighted correlations of exposure severity and posttraumatic stress symptoms.

Study	*N*	Age	% Women	% Minority	Months since Katrina	No. Exposure Items	PTSD Measure	*r*
Beaudoin (2009)	968	47.5	63.0	100	10	5	Breslau 7-item	.17
Cepeda et al. (2010)	350	33.9	37.0	98.6	15	3	NWS PTSD	.06
Cieslak et al. (2009)	90	41.6	33.3	NA	14	16	PCL-S	.39
Hirschel & Shulenberg (2009)	337	54.0	37.0	7.0	5	7	PCL-S	.21
Reuther et al. (2010)	609	20.7	72.7	18.0	3	24	IES-R	.35
Sprang & Lajoie (2009)	101	42.0	63.0	65.3	13	6	PCL-C	.40
Wadsworth et al. (2009)	93	44.0	58.1	65.6	4.5	46	UCLA PTSD	.19
Weems et al. (2007)	386	32.8	60.3	24.7	3.5	23	PTSD Checklist	.39

On average, the studies were conducted eight and a half months after Hurricane Katrina (median = 7.5, range = 3–15). Seven studies measured exposure with multiple binary questions and created a composite severity score. The remaining study [33] assessed exposure with 3 questions, including two binary and one 0 to 6 scale. They created an 8-point composite score using the sum of the scores. The number of exposure questions asked varied from 3 to 46, with an average of 16.3 (median = 11.5). The questions from each study are listed in Table 2. It should be noted that Wadsworth et al. [38] combined DRS and Life Event Questionnaire to measure exposure severity. Not only did this result in a relatively high (46) number of questions, it prevented the examination of DRS alone.

**Table 2:** Questions measuring exposure in each included study.

Study	No. of Questions	Items
Beaudoin (2009)	5	Presence in New Orleans when the hurricane hit; lost a job; home or apartment severely damaged; friend or relative die; friend or relative experienced other physical injury
Cepeda et al. (2010)	3	Felt like life was in danger; self or member of household injured; property damage
Cieslak et al. (2009)	16	Physically injured; physical danger; stranded; assaulted or raped; trapped; someone close died; home partially or completely destroyed; without food or water
Hirschel & Shulenberg (2009)	7	Felt safe during the hurricane; believed life was in danger; physical injuries; place of residence heavily damaged; took more than a month to return to normal commerce (e.g., shopping); lost job; took more than a month to return to work
Reuther et al. (2010)	24	TESS [13]
Sprang & Lajoie (2009)	6	Presence in the Gulf Coast region when the hurricane hit; perceived risk of harm to self; perceived risk of harm to loved ones; injury to self; family or friends injured, missing, or killed; exposure to other traumatic events since the hurricane
Wadsworth et al. (2009)	46	13-item hurricane exposure/loss assessing exposure to life-threatening events and loss, separation, and disruption+33-item Life Event Questionnaire [85].
Weems et al. (2007)	23	Separated from friends; separated from neighbors; separated from relatives; home damaged/destroyed; saw trees being damaged; heard about tornadoes in area; taken to different city/state; saw others hurt/sick/die; saw breaking windows/doors; separated from pets; witnessed crime or violence; got hurt or sick; saw roads washed away/flooding; separated from child; rescued; trapped in shelter

In terms of instrument for measuring PTS, two studies used the PTSD checklist – specific version (PCL-S) [48], one used the civilian version of the PCL (PCL-C) [49], one study used the UCLA PTSD Index [50], one study adapted the child PTSD checklist for their adult participants [51], one study used the National Women's Study PTSD module (NWS-PTSD) [52], one study used the Impact of Event Scale – Revised (IES-R) [53], and one study used a 7-item screening scale developed by Breslau, Peterson, Kessler, & Schultz [54]. Three samples consisted of participants who were no longer living in affected areas at the time of the study [33], [38], [37].

#### Exposure severity and PTS

**Figure 2:**
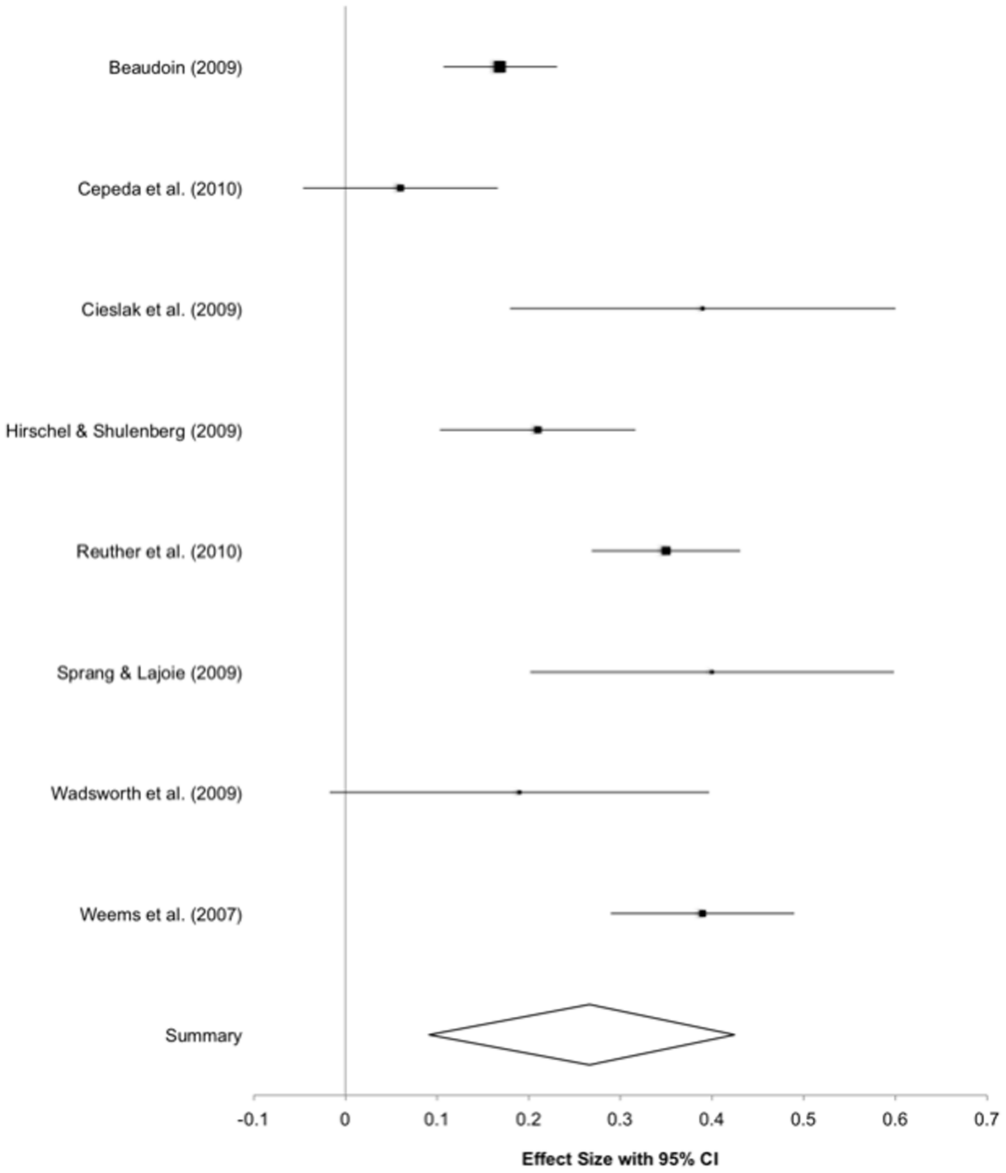
Forrest plot of constituent studies.

In the total set of eight samples, a significant correlation was found between severity of exposure and PTS. The ES ranged from *r* = .06 to .40. The combined sample-weighted ES was *r* = .266, *p*<.01, 95% CI [.173, .355] (Figure 2).

#### Test for heterogeneity

*Q* statistics yielded a significant result ( = 42.627, *p*<.001), indicating that there were differences in ESs beyond that expected due to sampling error alone. *I* ^2^ was high (83.6%), which showed that there was a high degree of true between-study variability. Because of the significant heterogeneity, we next tested the moderators using meta-regression to help identify between-study factors that might have contributed to the differences in effect sizes across studies.

#### Moderators

**Figure 3:**
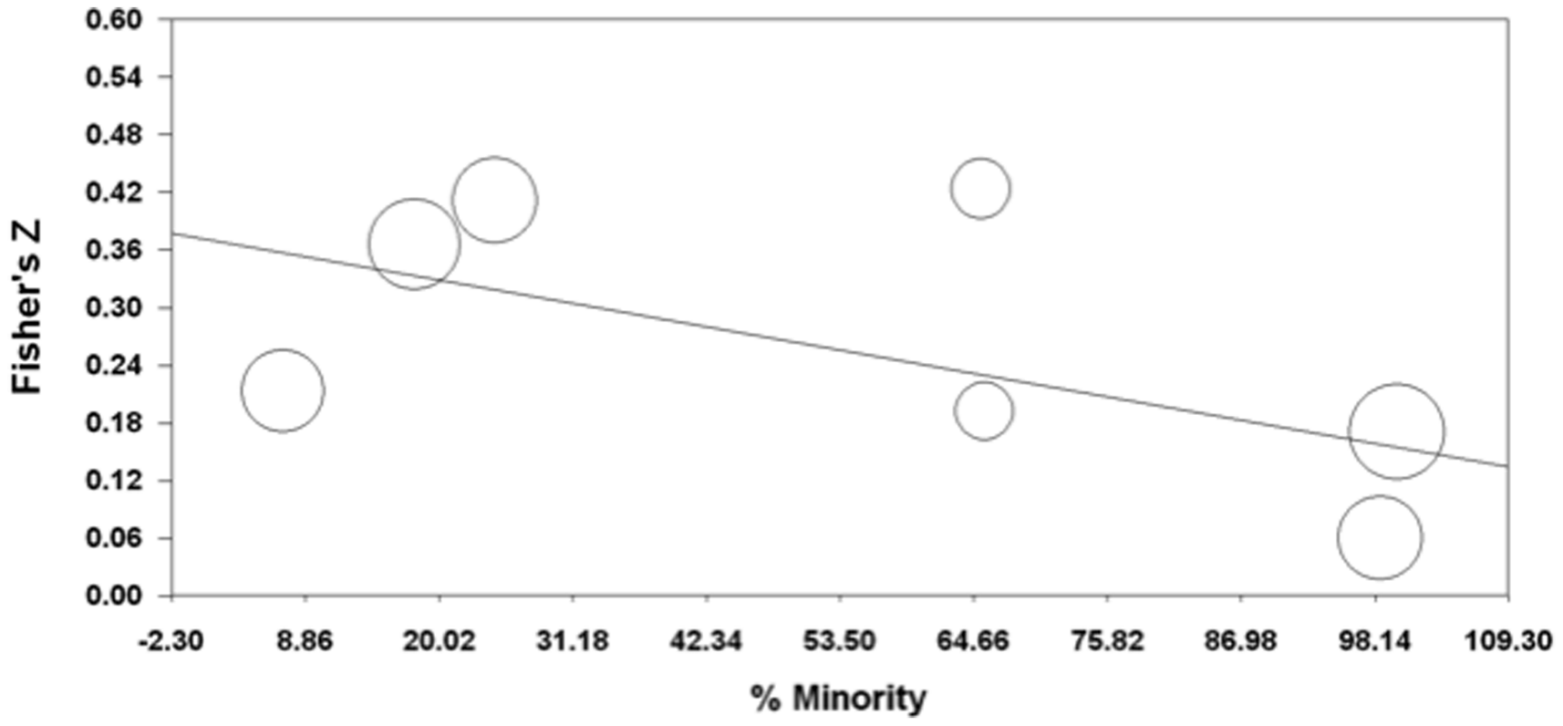
Meta-regression of studies' percentage of race/ethnicity minority on Fisher's Z, by maximum likelihood.

We tested the six moderators with six separate univariable simple mixed effects meta-regressions with ES as the dependent variable. Results are presented in Table 3. Of the six moderators, only percentage of racial and ethnic minorities in the sample was significantly associated with ES (Figure 3). Studies with a higher percentage of minority participants had smaller effect sizes. The number of exposure items became a significant moderator when an outlier, Wadworth et al. [38], was removed, *B* = .012, SE = .002, *p*<.001. No other significant relationships were found.

**Table 3:** Simple meta-regressions predicting effect size.

		Maximum Likelihood	
Predictor	*k*	*b*	*p*
% Women	8	.004	ns
Age	8	−.004	ns
% Minority	7	−.002	<.05
Months since Katrina	8	−.010	ns
No. Exposure Qs	8	.004	ns

*Note*. Models are random-effects weighted linear regressions calculated with weights equal to the reciprocal of the variance for each effect size plus a random-effects component.

#### Tests for publication bias

**Figure 4:**
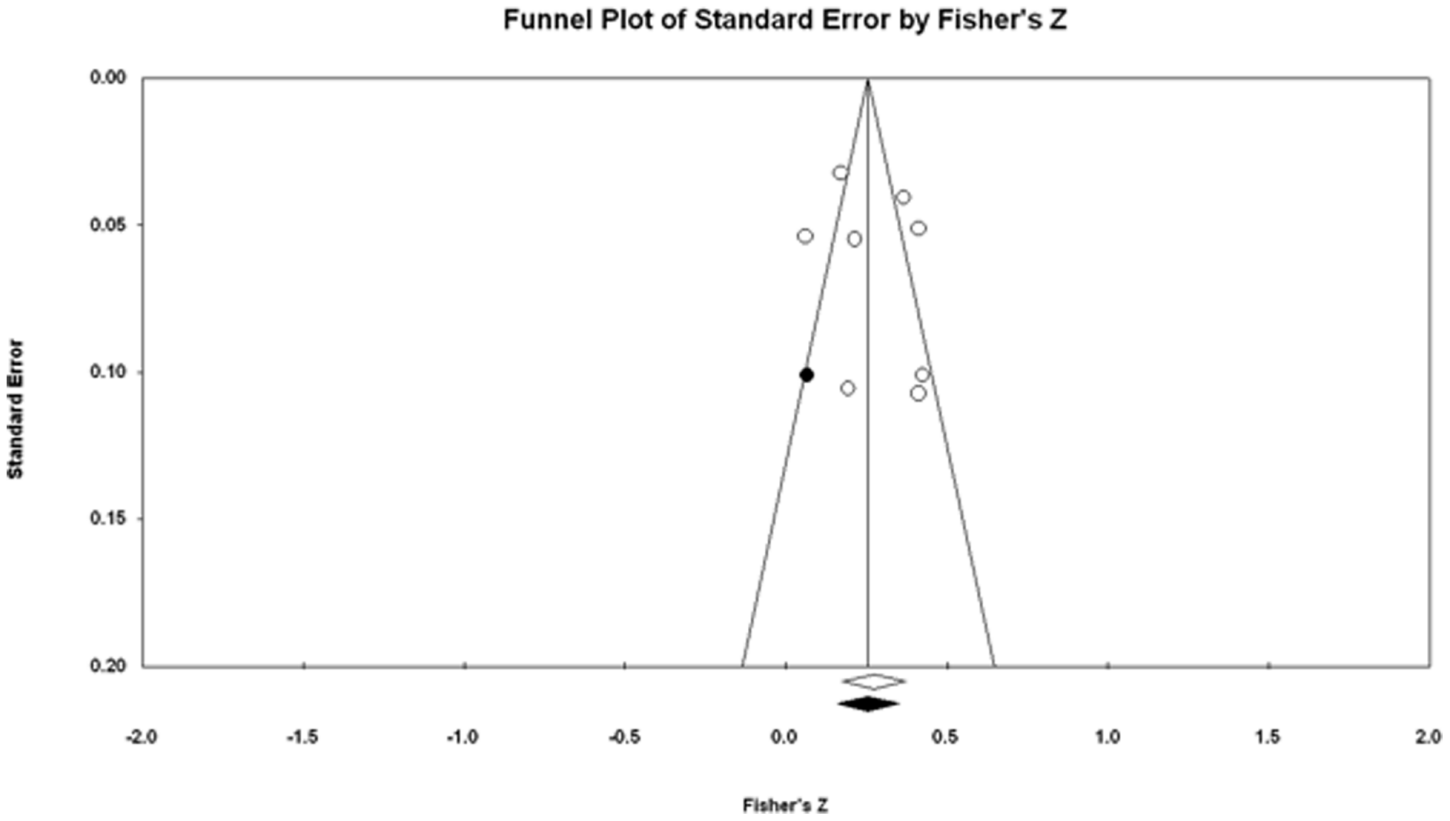
Exposure severity and posttraumatic stress: Funnel plot with imputed studies.

The funnel plot of the standard error against ES was not asymmetric by Egger's test, 1.580, 95% CI [−4.214, 7.375], *ns*. On the other hand, the trim-and-fill procedure suggested one additional study with a small ES to be filled in order to make the plot more symmetric (Figure 4). The addition of the imputed study yielded an adjusted effect of .251, 95% CI [.162, .336]. The relatively small change, along with the non-significant Egger's test, suggested that there was little evidence of publication bias.

### Discussion

In this meta-analysis, we examined the impact of severity of exposure to stressors on PTS among Hurricane Katrina survivors. The overall finding from eight samples (*N* = 2,934) of survivors of Hurricane Katrina confirmed the positive relationship between severity of exposure and PTS. The ES (*r* = .266) was in the small-to-medium range [41]. This finding was similar to results from previous meta-analyses on traumatic events in general (i.e., not specific to Hurricane Katrina or other natural disasters) [8], [9]. When compared with results based on civilian subsamples across an array of traumatic events, the ES from the present study was larger (vs., *r* = .18) [8]. The results of our meta-regression suggest that the discrepancy might be in part due to certain characteristics of the samples, especially the survivors' race and ethnicity. More generally, however, the discrepancy between our findings and previous ones may also be stem from the heterogeneity across disasters and disaster types. This remains an empirical question to be explored.

Even across primary studies of the same disaster and outcome, we found a high degree of heterogeneity in effect sizes. Interestingly, studies with higher percentage of White participants appeared to have larger effect sizes. This does not imply that minority groups were less impacted by the storm. Rather, it suggests that minority group members' PTS was less associated with the severity of exposure, which was operationalized as stressors directly related to Hurricane Katrina. Perhaps, in the context of poverty and systemic racism, minority groups have been exposed to more stressors before the storm, relative to their White counterparts [55] [56]. Through more frequent exposure to moderate and severe stressors, the more vulnerable survivors may have established a set of coping strategies that enabled them to more readily resist traumatic responses [24] [58] [57]. The effect of the stressors *directly* related to the storm might have been relatively attenuated by comparison. Nonetheless, that is *not* to say that ethnic minorities were not exposed to other stressors or risk factors that might have been exacerbated by Hurricane Katrina and its aftermath [18].

After the removal of an outlier that created a composite score of DRS and resource loss [38], the number of DRS measured was also a significant moderator. Studies that assessed more stressors had larger effect sizes. This might suggest that studies that cast a wider net might be more likely to find a stronger association between DRS and mental health outcomes. This points to a potential methodological problem in which the construct “exposure severity” varies between studies. One potential remedy might be to standardize the items included in the measure of exposure severity. Obviously, this solution is not without its limitations, given the between-disaster variability. The selection of items included might need to account for shared stressors (e.g., life loss, property loss) and stressors unique to a particular disaster (e.g., flooding in the case of a hydrological disaster). In many cases, researchers use exposure severity—typically a composite score—as a control variable. Limiting the items that constitute the composite score to common and shared stressors would permit better between-study comparisons.

#### Limitations

A number of limitations should be noted. First, the number of primary studies included in the meta-analysis was small (*k* = 8). The null results in the meta-regression for four of six variables may be due to lack of statistical power. Because power analysis in meta-analysis of studies with varying variance requires full covariance matrix [59], we were unable to perform it. One reason for the small sample size was that six additional studies that met the criteria of our literature search did not report the bivariate relationship between exposure severity and PTS, preventing us from calculating their effect sizes. Future studies should strive to adhere to the reporting guidelines of the American Psychological Association [60] on reporting bivariate relationships in order to facilitate meta-analytic procedures. Likewise, to the extent that researchers strive for cross-study consistency in measures of exposure and outcomes, comparisons will be more readily achieved.

Second, the generalizability of the current results is limited by the relatively narrow scope of our inclusion criteria. We focused only on Hurricane Katrina and only on PTS. By constraining the event, we removed some of the systematic sources of heterogeneity and improved the internal validity. We did not include other common psychological problems mostly because the number of studies that included additional psychological variables was even fewer. However, the results may not be generalizable to other disasters, which is a common limitation of disaster research. Building upon the current work, future meta-analytic studies can enlarge the scope to include studies of other natural disasters and outcome variables.

Last, and perhaps most significantly, the authors of all eight primary studies created a composite score with the various DRS. This method in effect treats each DRS with an equal weight. This may be problematic, as previous studies that examined DRS independently have found that some DRS are more predictive of mental health issues than others [6], [12]. Since the primary studies included different sets of DRS, albeit with some overlap (Table 2), this source of heterogeneity cannot be quantitatively tested. Without the bivariate relationship between each DRS and PTS, we cannot estimate and compare the differences in salience among DRS and thus cannot confidently infer which stressors contributed most strongly to PTS.

The heterogeneity in the constituent studies confirms important theoretical and methodological challenges in generalizing findings across individual primary studies. In light of this, Study 2 was conducted, using raw data drawn from multiple samples of survivors of Hurricane Katrina. This provided the opportunity to examine and compare the contribution of each DRS to mental health problems following a natural disaster.

## Study 2: Integrative Data Analysis

### Introduction

As demonstrated in Study 1, one source of between-study variability was the heterogeneity of the operationalization of exposure. Namely, in studies that included more disaster-related stressors (DRS) in their composite score of exposure severity there were stronger associations between exposure and PTS. It remains unclear, however, whether this was due to the mere number of events or whether studies with a more inclusive list of DRS also captured “key” stressors that other studies have failed to detect. Ideally, a checklist that measures exposure severity should encompass all relevant stressors, without being exhaustive and overly taxing for respondents. The two more commonly used hurricane stressors checklists, TESS [13] and HURTE [7] both consist of approximately twenty questions and hence might not always be feasible to use. Also, the development of TESS was based on exploratory factor-analysis approach, which, as argued by Netland [29], [30], is not appropriate. This is because the measurement model for event checklists is better conceptualized as a “causal-indicator” model, in which items (i.e., DRS) influences the construct (i.e., exposure), rather than the other way around, in which the indicators are conceptualized as effects of a latent construct. In other words, the extent of exposure to a disaster should be considered as a result of the various DRS and not the reverse. Moreover, neither scales examined the item-level relationship between DRS and outcomes. Without knowing the associations between specific DRS and psychological outcomes, the construction of a checklist would be based on heuristics, not empirical evidence. And in turn, an inadequate measure of exposure biases not only the measure of exposure severity itself, but also the estimation of other risk and protective factors. The establishment of a systematic assessment of disaster experiences has important research and clinical implications [5], [61]. Geared with information on the relative impact of specific disaster-related events, relief efforts and post-disaster clinical services may advance in efficiency, in terms of both identifying survivors who are at relatively higher risk for developing problems and addressing specific stressors accordingly. Drawing on integrative data analysis (IDA) of raw data from multiple samples of Hurricane Katrina survivors, Study Two was designed to examine the associations between specific DRS and PTS as well as general psychological distress (GPD).

For the most part, disaster researchers have created composite exposure scores from positively endorsed DRS items. Although conventional, this practice assumes equal weighting of the DRS; each stressor is treated equally and assumed to be equally predictive of the outcomes of interest. Because exposure severity is often included as a covariate, the item-level association with mental health outcomes are seldom reported (for exceptions, see Goenjian et al. [28]; Heir & Weisæth [6]). The omission of the bivariate relationship between each DRS and PTS, in turn, biases the estimation and limits the comparability of the impact among different DRS on post-disaster psychopathology.

A relatively large number of studies have been conducted on Hurricane Katrina, which provides an apt opportunity to integrate and compare results from different samples. The goal of the current IDA was to examine and compare the effect sizes of different DRS in the context of Hurricane Katrina.

#### Integrative data analysis

IDA is a method of simultaneously analyzing multiple independent samples [62]. Its function and goals are similar to traditional meta-analysis, in which aggregated parameter estimates (i.e., effect sizes) are combined. What differs is that IDA utilizes actual raw data from existing studies. The advantage is that models can be re-specified to fit the need of emerging research questions, such as the current one. Moreover, larger sample sizes result in increased statistical power and greater potential for generalizability due to greater sample heterogeneity. Nonetheless, IDA has a number of methodological challenges, such as the need to account for historical and regional effects as well as sample heterogeneity across studies [62]. Because the current study drew on data that came from residents of the same geographic area (New Orleans metropolitan area) after the same disaster (Hurricane Katrina), the historical and regional effects on the variability in outcome variables can be assumed to be minimal. Any variability found can be more confidently attributed to between study and sample differences.

In this study, the relationship between DRS and mental health, indicated by PTS and general psychological distress (GPD), were examined using a pooled sample from multiple studies of survivors of Hurricane Katrina.

### Methods

#### Literature Search

A literature search similar to the one described in Study 1 was conducted to identify eligible studies. The difference is that in Study 2 we did not screen out studies that did not report the measure of exposure severity, PTS, or GPD as continuous variables. This resulted in 21 independent studies that included an adult (age 18+) sample. Authors and research groups of all 21 studies were contacted to obtain raw data on mental health outcome and measure of exposure severity, in addition to basic demographic variables. Of the 21, two research groups rejected the solicitation, six never responded, four agreed to share the data but have not done so by the time of the data analysis, and nine provided the data ([12], [34], [35], [63]–[66], [70]–[71]). Of the two rejections, one was on the basis of an overlapping project and the other was due to the Principal Investigators' ongoing use of the dataset.

#### Samples. Posttruamtic Stress

PTS was measured in eight of the nine obtained datasets. Among them, seven studies used a standardized self-report measure and one study used a clinician-administered measure (CIDI). The latter study was excluded from the current analysis because PTS was reported as a binary diagnosis (PTSD), without a measure of its severity.

Four different self-report measures were represented across the seven remaining studies. Four studies employed the PTSD checklist (PCL) [48], [67] and the other three studies used the Impact of Events Scale–Revised (IES-R) [53], the Trauma Screening Questionnaire (TSQ) [68], and the PTSD Symptom Scale Self-Report (PSSSR) [69], respectively. To maximize comparability, the four samples that reported PTS using the PCL were included in the current study: Hirschel & Shulenberg [35]; Cieslak et al. [34]; McLeish & Del Ben [63]; and LaJoie, Sprang, & McKinney [64].

#### GPD

Four of the nine studies included a self-report measure of GPD. Three studies used the K6 [70] and one study administered the Quality of Well­Being Self­Administered (QWB­SA) [71]. Two of the three studies that employed the K6 along with various DRS were included in the analysis: Hurricane Katrina Community Advisory Group Study (HKCAG) [12], [65] and the Resilience In Survivors of Katrina study (RISK) [66]. To further ensure comparability of the two samples, the participants of the two studies who resided within the metropolitan area of New Orleans before Hurricane Katrina were selected. This yielded 594 participants from the HKCAG study (57.0% of original sample) and 354 participants from the RISK study (88.1% of original sample).

#### Measures. PTSD

Symptoms of PTSD were measured in the four included samples (Table 4) using the PCL. The PCL is a 17-item, 5-point Likert-type self-report measure. Each of the 17 items directly corresponds to one of the PTSD diagnosis criteria of the *DSM IV* [72]. Respondents rated the severity of each symptom over the past 30 days. A severity score (range = 17 to 85) was created by summing scores on each item. Researchers have reported strong psychometric properties, including high internal consistency, convergent validity, and diagnostic efficiency across different populations [48], [73], [74]. A four-factor model [75] was tested using multi-group confirmatory factor analysis (CFA), in order to ensure measurement invariance (reported below).

**Table 4:** Descriptions of studies.

	Study	PIs	Timing of Study	*N*	Sample	Exposure Measure	Outcome Measure
1	Hirschel & Shulenberg	Swanson	Jan 2006	399	Community	7 dichotomous items	PCL
2	Cieslak, Benight et al.	Kissinger	Oct 2006	90	Student	16 dichotomous items	PCL
3	McLeish & Del Ben	McLeish	Sep 2005	76	Clinical	Various Likert Scales	PCL
4	LaJoie et al.	LaJoie	Sep 2006	101	Community	4 items (3 dichotomous)	PCL
5	Hurricane Katrina Community Advisory Group	Kessler, Galea et al.	Jan–Mar 2006	1043	Community	10 dichotomous items	K6
6	Resilience In Survivors of Katrina (RISK) study	Paxson, Rhodes, Waters	May 2006–Mar 2007	402	Student	13 dichotomous items	K6

#### General Psychological Distress

The K6 scale of nonspecific psychological distress [70] was used to assess DSM–IV mood and anxiety disorders within the previous 30 days. The K6 scale has been shown to have good psychometric properties [76] and has been used in previous research on the psychological functioning of Hurricane Katrina survivors [12], [66]. It includes items such as “during the past 30 days, about how often did you feel so depressed that nothing could cheer you up?” Respondents answered on a 5-point rating scale ranging from 0 = “none of the time” to 4 “all the time”. Scale scores range from 0 to 24. A previous validation study [77] suggests that a scale score of 0–7 can be considered as probable absence of mental illness, a score of 8–12 can be considered as probable mild or moderate mental illness (MMI), and a score of 13 or greater can be considered as probable serious mental illness (SMI). Rhodes et al. [66] reported that the Cronbach's alpha of the K6 scale in the RISK study was *α* = .80. Although no internal consistency was reported from the HKCAG studies, past epidemiological studies reported a similar level of internal consistency (*α* = .89) [77].

#### Exposure Severity

A large degree of variability was found in the DRS included in the six studies. Only DRS that are directly related to the physical nature of the disaster were included; stressors such as loss of job were excluded from the current analyses. The range of DRS included both objective events (e.g., lacked food or water, injury) and subjective appraisals (e.g., fear). Delayed evacuation, defined as leaving the region during or after the storm, was coded and included in the analysis as a proxy of other unaccounted DRS. All the included DRS are reported in Table 5.

**Table 5:** Disaster-related stressors assessed in studies.

Study	Danger	Delayed evacuation	Fear	Injury	Home damage	Property damage	Family injured	Lacked food or water	Separated from family	Death	Strand	Trapped	Media exposure	Power outage	Child safety uncertain	Family safety uncertain	Pet loss	Lacked medication or medical care	Exposed to toxins	Witnessed death	Witnessed drowning
Hirschel & Shulenberg	X	X	X	X	X																
Cieslak et al.	X	X	X	X	X	X	X	X	X	X	X	X									
McLeish & Del Ben					X			X		X			X	X							
LaJoie et al.		X	X				X														
HKCAG			X	X				X		X					X	X	X	X	X	X	X
RISK		X			X	X		X		X					X	X	X	X			

*Note*. CAG = Hurricane Katrina Community Advisory Group; RISK = Resilience in Survivors of Katrina.

#### Demographic Variables

Age, gender, race and ethnicity were included in the current study. Gender was dummy-coded as 1 = “female” and race/ethnicity was dummy coded as 1 = “White.” Because studies did not consistently record date of interview, time since Hurricane Katrina was not included in the analyses.

#### Statistical Analysis

Before data from multiple studies can be analyzed collectively, measurement invariance must be established by demonstrating that the outcome measures reflect the same construct (i.e., PTS and GPD) [62]. It is when the definition and measurement of constructs agree across studies that IDA becomes possible [62]. Confirmatory factor analyses were conducted using M*plus* 6 for both PCL and K6. For PCL, a four-factor model (re-experiencing, avoidance, numbing, and arousal) was specified [75], whereas a one-factor model was specified for K6.

Linear and logistic regression analyses were conducted to estimate the association between DRS and symptoms of PTSD and GPD. All analyses were conducted using *R*.

### Results

#### Posttraumatic Stress

In order to determine if the different samples could be combined, measurement invariance was evaluated with the following steps: (1) configural model assessment, (2) test of equal factor loadings (i.e., weak measurement invariance), and (3) test of equal intercepts (i.e., strong measurement invariance). This procedure involves testing a series of nested models with a less restricted model compared to a more restricted model (i.e., more degrees of freedom). To assess the significance of each comparison, we evaluated if (a) the RMSEA value of the nested model fell within the RMSEA confidence interval of the comparison model [78] and (b) the change in CFI was ≤.01 [79]. A value beyond these specifications suggests that the imposed restrictions are not supported. A four-factor model was used based on previous psychometric studies of the PCL [75]. As shown in Table 6, we failed to establish configural invariance with the four studies included in the current analysis. Nonetheless, when the sole clinical sample [63] was removed, strong factorial invariance was established across the remaining three studies. These three samples were thus combined as one pooled sample for the remaining analyses.

**Table 6:** Fit indices for the nested sequence in the multiple group confirmatory factor analysis on PCL.

	Model	*χ^2^*	*Df*	*p*	RMSEA	RMSEA 90% CI	CFI	ΔCFI	TLI	ΔTLI	Pass?
4 studies	Configural Invariance	1499.145	536	<.001	.105	.099–.111	.889		.887		Fail
3 studies	Configural Invariance	942.616	365	<.001	.094	.087–.101	.919		.910		Pass
	Loading Invariance	972.167	365	<.001	.092	.085–.100	.921	.003	.912	.002	Pass
	Intercept Invariance	1071.692	391	<.001	.095	.088–.101	.912	.009	.908	.004	Pass

The pooled sample size was 647. Within the three samples, the PCL scores were *M* = 34.47 (*SD* = 15.89), 40.88 (*SD* = 18.22), and 49.23 (*SD* = 20.51), respectively. The average PCL score across the three samples was *M* = 38.47 (*SD* = 18.13). The correlation between the DRS, PCL, and demographic variables are presented in Table 7.

**Table 7:** Zero-order correlation matrix for variables included in the PCL analyses.

		*1*	*2*	*3*	*4*	*5*	*6*	*7*	*8*	*9*	*10*	*11*	*12*	*13*	*14*	*15*	*16*
1	Age	-															
2	Female	−.01	-														
3	White	.36***	.01	-													
4	Danger	−.01	−.03	.08	-												
5	Delayed Evacuation	.06	−.09*	−.09*	.09*	-											
6	Fear	−.09*	−.01	−.17***	.22***	.34***	-										
7	Injury	.05	.01	.05	.15***	.03	.03	-									
8	Home Damage	.08	.09*	.08	.01	−.01	.09*	.03	-								
9	Property Damage	−.04	.19*	−.25**	.10	−.09	.19*	.05	.71***	-							
1	Family Injury	.07	.09	.05	.03	.41***	.45***	.03	.04	.13	-						
11	Lacked Food or Water	.05	.01	−.05	.20*	.43***	.26**	.19*	.16	.21*	.24**	-					
12	Separated from Family	−.12	.06	−.23**	.10	.28***	.16	.10	.19*	.16	.19*	.16	-				
13	Death	−.07	−.02	−.02	.22**	.08	.16	.11	.18*	.19*	.36***	.12	.26**	-			
14	Stranded	.09	−.09	.15	.26**	.40***	.25**	.15	.14	.13	.15	.59***	.14	.17*	-		
15	Trapped	.06	.01	−.06	.27***	.35***	.33***	.37***	.10	.11	.17*	.41***	.16	.12	.58***	-	
16	PTSD	−.07	.10*	−.15***	.02	.10*	.30***	.09*	−.02	.10	.28***	.24**	.22**	.27**	.27**	.13	-
	Mean (SD) or %	46.77 (19.48)	58.02	58.89	9.89	49.15	31.38	8.04	89.51	73.79	47.76	32.41	65.52	2.69	31.03	2.69	38.47
	*n*	647	617	647	647	647	615	647	515	145	245	145	145	145	145	145	18.13

NOTE:

*p<.05,

**p<.01,

***p<.001.

Two linear regression models predicting PCL were estimated using DRS as predictors and demographic variables (age, gender, and race) as covariates (Table 8). In Model 1, data from all three studies that measured PCL were included. Only two DRS were shared across all three studies: delayed evacuation and fear. The results indicated that fear but not delayed evacuation was predictive of PCL, *β* = .269. Model 2 included data from Hirschel & Shulenberg [35] and Cieslak et al. [34], with delayed evacuation, fear, injury, and home damage included as predictors. The results indicated that fear (*β* = .166) and injury (*β* = .153) were predictive of symptoms of PCL.

**Table 8:** Unstandardized and standardized coefficients of two linear regression model predicting symptoms of PTSD (PCL).

	B	SE	*t*	*β*	*p*	
*Model 1*						
Female	4.341	1.488	2.916	.107	.004	**
White	−6.447	1.384	−4.657	−.104	<.001	***
Age	.005	.049	.108	−.010	.914	
Delayed evacuation	−.479	1.571	−.305	.018	.761	
Fear	1.881	1.695	6.420	.269	<.001	***
*Adj R^2^*	.132***					
*Model 2*						
Female	2.936	1.646	1.784	.065	.075	
White	−6.464	1.600	−4.041	−.080	<.001	***
Age	−.045	.052	−.855	−.044	.393	
Delayed evacuation	−2.032	1.669	−1.218	−.035	.224	
Fear	8.347	1.991	4.193	.166	<.001	***
Injury	9.722	2.849	3.413	.153	.001	**
Home damage	−3.643	4.539	−.803	−.039	.423	
*Adj R^2^*	.098***					

Note.

**p<.01,

***p<.001.

#### General Psychological Distress

We were unable to establish factorial invariance across the two samples that measured GPD with K6. In fact, the one-factor model did not hold in either sample, suggesting that the one-factor specification might not be accurate. Because of the lack of measurement invariance, bivariate relationships (odds ratio and relative risk) are reported separately for the two samples (Table 9).

**Table 9:** Bivariate association between disaster-related stressors and serious mental illness (K6≥13) across two studies.

	HKCAG					RISK				
DRS	*n*	Caseness	*OR*	*RR*	*p*	*n*	Caseness	*OR*	*RR*	*p*
Child safety	410	12	1.611	1.508	ns	354	22	2.622	2.217	<.01
Family safety	412	39	1.764	1.655	ns	353	40	.882	.899	ns
Pet loss	582	17	3.182	2.660	<.001	346	15	2.186	1.894	<.05
Death	582	23	3.947	3.185	<.001	352	23	2.466	2.115	<.01
Lacked food, water, or clothes	577	53	3.691	3.290	<.001	354	27	1.968	1.061	<.05
Lacked medication or medical care	574	38	2.771	2.464	<.001	354	32	2.461	2.151	<.01
Injured	579	22	6.563	4.557	<.001					
Exposed to toxins	560	26	3.177	2.717	<.001					
Danger	63	2	.933	.946	ns					
Witnessed death	579	17	2.435	2.148	<.01					
Witnessed drowning	579	27	2.888	2.513	<.001					

Note. CAG = Hurricane Katrina Community Advisory Group; RISK = Resilience in Survivors of Katrina.

The pooled sample size was 948. The pooled average K6 score across the two samples was *M* = 7.49 (*SD* = 4.39). The average K6 score of the two studies was *M* = 6.77 (*SD* = 5.27) and *M* = 7.93 (*SD* = 3.70), respectively. The correlation between the DRS, K6, and demographic variables are presented in Table 10.

**Table 10:** Zero-order correlation matrix for variables included in the analyses predicting general psychological distress (GPD).

		*1*	*2*	*3*	*4*	*5*	*6*	*7*	*8*	*9*	*10*	*11*	*12*	*13*	*14*	*15*	*16*	*17*
1	Age	-																
2	Female	−.33***	-															
3	White	.49***	−.29***	-														
4	Child safety	.08*	.00	−.07	-													
5	Family safety	−.20***	.12**	−.15***	.00	-												
6	Property loss	−.03	.01	−.13*	.06	.06	-											
7	Vehicle loss	.06	.02	−.15**	.08	.09	.20***	-										
8	Pet loss	−.11***	.02	−.08*	.05	.12***	.05	.04	-									
9	Death	−.11***	.12***	−.19***	.03	.11**	.05	.13*	.15***									
10	Lack food, water, or clothes	.08*	−.03	.05	.15***	.07	.07	.07	−.03	.06	-							
11	Lack medication or medical care	−.05	.02	−.05	.14***	.16***	.09	.07	.05	.20***	.30***	-						
12	Injured	−.04	.04	−.10*	.17***	.01	NA	NA	.11**	.11**	.19***	.21***	-					
13	Exposed to toxins	−.05	−.04	−.04	.07	.00	NA	NA	.09*	.12**	.17***	.18***	.24***	-				
14	Threatened	.06	−.15	.03	−.10	.11	NA	NA	−.26*	−.03	.13	.24	.23	−.02	-			
15	Witnessed death	−.24***	−.03	−.09*	.05	.07	NA	NA	.08*	.09*	.11**	.06	.09*	.18***	.42***	-		
16	Witnessed drowning	−.09*	.07	−.16***	.05	.05	NA	NA	.12**	.13**	.19***	.07	.15***	.11**	−.08	.09*	-	
17	GPD	.05	.07*	.02	.09*	.02	.14**	−.01	.18***	.18***	.23***	.22***	.32***	.24***	−.01	.15***	.19***	-
	Mean (SD) or %	40.78 (16.17)	70.15	47.6	20.6	72	92.5	46.2	14	19.7	53	40.1	10.7	22	17.5	14.7	23.2	7.49 (4.39)
	*n*	948	948	934	770	771	346	346	940	946	943	940	590	569	63	591	591	936

*Note*. NA = not available;

*p<.05,

**p<.01,

***p<.001.

A linear regression model predicting K6 was estimated using DRS as predictors and demographic variables (age, gender, and race) as covariates. The results indicated that pet loss; death of a family member or friend; lacking food, water, or clothing; and lacking medication or medical care were all predictive of GPD (Table 11). The standardized estimates ranged from *β* = .116 (lacked medication or medical care) to *β* = .200 (pet loss).

**Table 11:** Unstandardized and standardized coefficients of a linear regression model predicting general psychological distress (K6).

	B	SE	*t*	*β*	*p*	
Age	.024	.013	1.875	.078	.061	
Female	1.065	.403	2.643	.098	.008	**
White	.554	.382	1.450	.060	.147	
Child safety	.248	.283	.874	.031	.383	
Family safety	−.263	.365	−.721	−.026	.471	
Pet loss	2.536	.449	5.652	.200	<.001	***
Death	1.318	.399	3.301	.120	.001	**
Lack food, water, or clothes	1.514	.338	4.477	.165	<.001	***
Lack medication or medical care	1.073	.347	3.091	.116	.002	**
*Adj R^2^*	.129***					

*Note.*

**p<.01,

***p<.001.

### Discussion

In their review of the literature, Norris and Wind [10] identified loss of life, bereavement, threat to life, injury, and fear, and witnessing of horror as potentially the most traumatic aspects to a disaster. Our findings, drawn from five independent samples of Hurricane Katrina survivors, are generally consistent with their conclusions. In particular, we found that, among different primary, disaster-related stressors, threat to physical integrity of self and others had the strongest association with posttraumatic stress (PTS) and general psychological distress (GPD). Furthermore, the lack of basic necessities, such as food, water, and medical care, and loss of pet were also found to be strongly associated with both PTS and GPD.

In this study, we included DRS at the item level to estimate each stressor's unique contribution to mental health. We included all DRS that were available in each dataset, both subjective and objective stressors. From the three studies that included PCL as a measure of PTS, our pooled results suggest that fear was the most consistent predictor of symptom severity. The effect size of experiencing intense fear dropped from .27 to .17 when one study was removed and physical injury was added as a predictor. Physical injury had a similar effect size, *β* = .15, suggesting that both subjective and objective threat to one's integrity are associated with PTS.

This set of results was augmented by the inclusion of two samples with K6, a measure of general psychological distress. Our results suggest that a lack of basic necessities during the storm was associated with higher levels of psychological stress. In particular, the lack of medication or medical care, as well as food and water, can be interpreted as a source of threat to one's well being. Consistent with past finding [3], bereavement was also associated with psychological distress. Notably, a strong association between pet loss and GPD was found in both samples. At the bivariate level, pet loss was associated with 2- to 3-fold increase in odds of having a serious mental illness. Once demographic variables and other DRS were accounted for, the loss of pet was associated with a 2.5 points increase on the K6 (range = 0 to 24). The impact of pet loss is understudied but given the current findings, which is consistent with the few past studies on the topic [80], [81], it should perhaps be included in future disaster studies.

#### Limitations

To our knowledge, this study is the first to integrate multiple samples from the same disaster to form a larger dataset. The advantage of analyzing multiple samples, especially in the context of studying the impact of disasters, includes the opportunity to survey a larger range of DRS and higher statistical power. On the other hand, although an explicit effort was made to include as many studies and stressors as possible, the relative lack of between-study overlap in stressor inclusion and outcome measures made it challenging to conduct meaningful cross-study comparison. In particular, the relative lack of overlap in DRS across the three PSTD studies limited the number of stressors we were able to examine in the multivariate models.

Relatedly, it should be noted that we only included primary DRS and omitted secondary and chronic ones, such as financial, occupational, and marital stressors. Studies have found that these day-to-day stressors and chores can be more distressful in the long-run [82].

Another major limitation of the current study was that the studies included in the current IDA were cross-sectional in design; only one [66] had pre-disaster measures of mental health. Without baseline levels, the estimation of the impact of disaster exposure, even multiple samples were pooled, would likely be biased. The vast majority of studies of disaster outcomes lack pre-disaster data [83]. Pre-disaster data allow researchers to better clarify the temporal order of the event and outcome variables, as well as to control for pre-existing levels of psychological health. Also, the DRS included in the current IDA were all based on self-report, which may be susceptible to subjective biases. DRS, even the ones based on objective events, might have been influenced by the respondent's post-disaster mental health, thus confounding the present findings. Compared with objective measures of trauma, however, subjective experiences of events might be more predictive of psychological functioning [84].

## General Discussion

The results from the meta-analysis of eight primary studies of Hurricane Katrina (Study 1) confirmed that there was a small-to-medium positive relationship between exposure severity and PTS. Moreover, the between-study heterogeneity in the magnitude of this relationship was partially explained by two sample and study factors, namely proportion of minority participants and number of questions asked about DRS. The latter was particularly relevant to the current project, as it suggest that the operationalization of exposure severity can likely affect (and bias) the estimations of other predictors.

What remained unclear, however, was what exactly drove this moderating relationship. Given that all the primary studies included in the meta-analysis created an exposure severity composite score using varying DRS, in many ways the construct itself was not identical across the studies. This was the motivation behind Study 2, in which DRS were examined at the item-level (vs. composite score) across five samples of Hurricane Katrina survivors. The results confirmed that specific events, such as injury or pet loss, as well as subjective perception of threat to the physical integrity of oneself and others were predictive PTS and GPD. Basic necessities, such as the lack of food, water, medicine, and medical care were also robust predictors. These findings reinforce the importance of providing necessities and medical care, as well as accommodation for pets, if possible, in the aftermath of a disaster. The results of this study shed light on to the current lack of consensus regarding the items (and number of items) of DRS to be included in a measure of exposure severity. As discussed earlier, perhaps researchers, when using the construct as a control variable, should include in the composite score the DRS that are generally found across studies to be robust in predicting outcome variables (e.g., PTS or GPD). A more or less standardized measure of exposure can facilitate better cross-study comparisons and, in turn, the generalizability of research findings.

In looking at the data reported in the published reports we examined, we recommend that future studies strive to include bivariate associations to facilitate systemic review and meta-analysis. Our findings indicate that they should also consider using composite scores of exposure severity with caution, given that there is great variability in the impact of each DRS. It might be advisable to separate different types of DRS and to include relatively understudied but evidently significant stressors such as pet loss. The use of objective measures might also help complement the subject self-report events.

As noted by Norris and Wind, “exposure to disaster is an inherently complex, multifaceted phenomenon” [10, p. 29]. The above studies provided evidence that a wide range of experiences can potentially affect post-disaster mental health. They represent a first step in identifying cross-cutting issues of relevance to the assessment of DRS. Given the importance of exposure severity in the impact of disasters, it is surprising that relatively little attention has been paid to the ways in which it is operationalized. The current study is but one of many possible ways to help begin to untangle this issue.

## Supporting Information

Checklist S1 **PRISMA Checklist.**
(PDF)
